# Evaluation of tumor hypoxia prior to radiotherapy in intermediate-risk prostate cancer using ^18^F-fluoromisonidazole PET/CT: a pilot study

**DOI:** 10.18632/oncotarget.24234

**Published:** 2018-01-13

**Authors:** Stéphane Supiot, Caroline Rousseau, Mélanie Dore, Catherine Cheze-Le-Rest, Christine Kandel-Aznar, Vincent Potiron, Stéphane Guerif, François Paris, Ludovic Ferrer, Loïc Campion, Philippe Meingan, Gregory Delpon, Mathieu Hatt, Dimitris Visvikis

**Affiliations:** ^1^ Institut de Cancérologie de l'Ouest, Bld Jacques Monod, 44805 Nantes-Saint Herblain, France; ^2^ Centre de Recherche en Cancérologie Immunologie Nantes/Angers (CRCNA, UMR 1232 INSERM), Institut de Recherche en Santé de l'Université de Nantes, 44007 Nantes CEDEX 1, France; ^3^ Centre Hospitalier Universitaire, 86021 Poitiers, France; ^4^ Centre Hospitalier Universitaire, 44000 Nantes, France; ^5^ Laboratoire de Traitement de l'Information Médicale (LaTIM), INSERM, UMR 1101, IBSAM, UBO, UBL, IBRBS, Faculté de Médecine, 29238 Brest, France

**Keywords:** hypoxia, prostate cancer, misonidazole, FAZA, HIF

## Abstract

**Purpose:**

Hypoxia is a major factor in prostate cancer aggressiveness and radioresistance. Predicting which patients might be bad candidates for radiotherapy may help better personalize treatment decisions in intermediate-risk prostate cancer patients. We assessed spatial distribution of ^18^F-Misonidazole (FMISO) PET/CT uptake in the prostate prior to radiotherapy treatment.

**Materials and Methods:**

Intermediate-risk prostate cancer patients about to receive high-dose (>74 Gy) radiotherapy to the prostate without hormonal treatment were prospectively recruited between 9/2012 and 10/2014. Prior to radiotherapy, all patients underwent a FMISO PET/CT as well as a MRI and ^18^F-choline-PET. ^18^F-choline and FMISO-positive volumes were semi-automatically determined using the fuzzy locally adaptive Bayesian (FLAB) method. In FMISO-positive patients, a dynamic analysis of early tumor uptake was performed. Group differences were assessed using the Wilcoxon signed rank test. Parameters were correlated using Spearman rank correlation.

**Results:**

Of 27 patients (median age 76) recruited to the study, 7 and 9 patients were considered positive at 2.5h and 3.5h FMISO PET/CT respectively. Median SUV_max_ and SUV_max_ tumor to muscle (T/M) ratio were respectively 3.4 and 3.6 at 2.5h, and 3.2 and 4.4 at 3.5h. The median FMISO-positive volume was 1.1 ml.

**Conclusions:**

This is the first study regarding hypoxia imaging using FMISO in prostate cancer showing that a small FMISO-positive volume was detected in one third of intermediate-risk prostate cancer patients.

## INTRODUCTION

Intermediate-risk prostate cancer is a highly heterogeneous disease with biochemical relapse occurring in 10 to 50 % of patients after ten years [1]. Patients with intermediate prostate cancer are routinely offered radical prostatectomy or radiotherapy and/or androgen depriving therapies, but when patients have the choice between the two options, no clinical or biological parameters are known to be useful in predicting which patients might be suitable or unsuitable candidates for surgery or radiotherapy, to better personalize their treatment decisions [2].

Hypoxia may help discriminate between standard-risk and aggressive prostate cancer based on histological analysis of markers in the prostatectomy specimen [3] or hypoxic signature combined with genomic instability [4]. Moreover, hypoxia is a major factor in prostate cancer radioresistance [3, 5, 6] inhibiting the production of reactive oxygen species during irradiation or by selecting radioresistant cells. It might therefore be necessary to avoid radiotherapy in hypoxic patients or combat hypoxia by increasing the dose of radiotherapy to small hypoxic sub-volumes using “dose-painting” strategies [7, 8], or to combine irradiation with hypoxia-modifying drugs [9].

Because biopsy sampling may miss areas of significant hypoxia, it is difficult to orientate patients in a hypoxia-based personalized approach based only on partial morphological analysis. Functional imaging of hypoxic regions within the prostate may help to address this. Among several radiotracers, ^18^F-fluoromisonidazole (FMISO) is one of the most widely studied [10]. Based on a small study where FMISO uptake was detected in 3 out of 4 prostate cancer patients at various stages [11], we hypothesized that FMISO can be detected in 75% of intermediate-risk prostate cancer patients and may therefore represent a non-invasive tool to map hypoxia within the whole prostate. To test this hypothesis, we assessed spatial distribution of ^18^F-fluoromisonidazole (FMISO) PET/CT uptake in the prostate prior to radiotherapy treatment in intermediate-risk prostate cancer patients and compared these results with anatomical imaging and Glut1 staining on biopsy specimens.

## RESULTS

### Patient characteristics

We recruited 27 patients (median age 76 [range 58–81]) with intermediate-risk prostate cancer between 09/2012 and 10/2014 (Supplementary Table 1A and 1B). Median PSA was 7.97 ng/ml (range 2.2–19 ng/ml). According to the Zumsteg classification [1], 19 (70.4 %) were considered as unfavorable intermediate-risk prostate cancer with at least one risk-factor. Three patients had contra-indications to MRI. Among the 24 others, a PI-RADS 4 or 5 tumor was detectable in 20 patients. The mean GTV MRI volume was 6 cm^3^ (range 3.4–11.2). One patient was unable to have a FCH PET for technical reasons. Among the 26 others, the mean GTV FCH volume was 11 cm^3^ (range 4–16).

### FMISO PET/CT prior to radiotherapy

**Figure 1:**
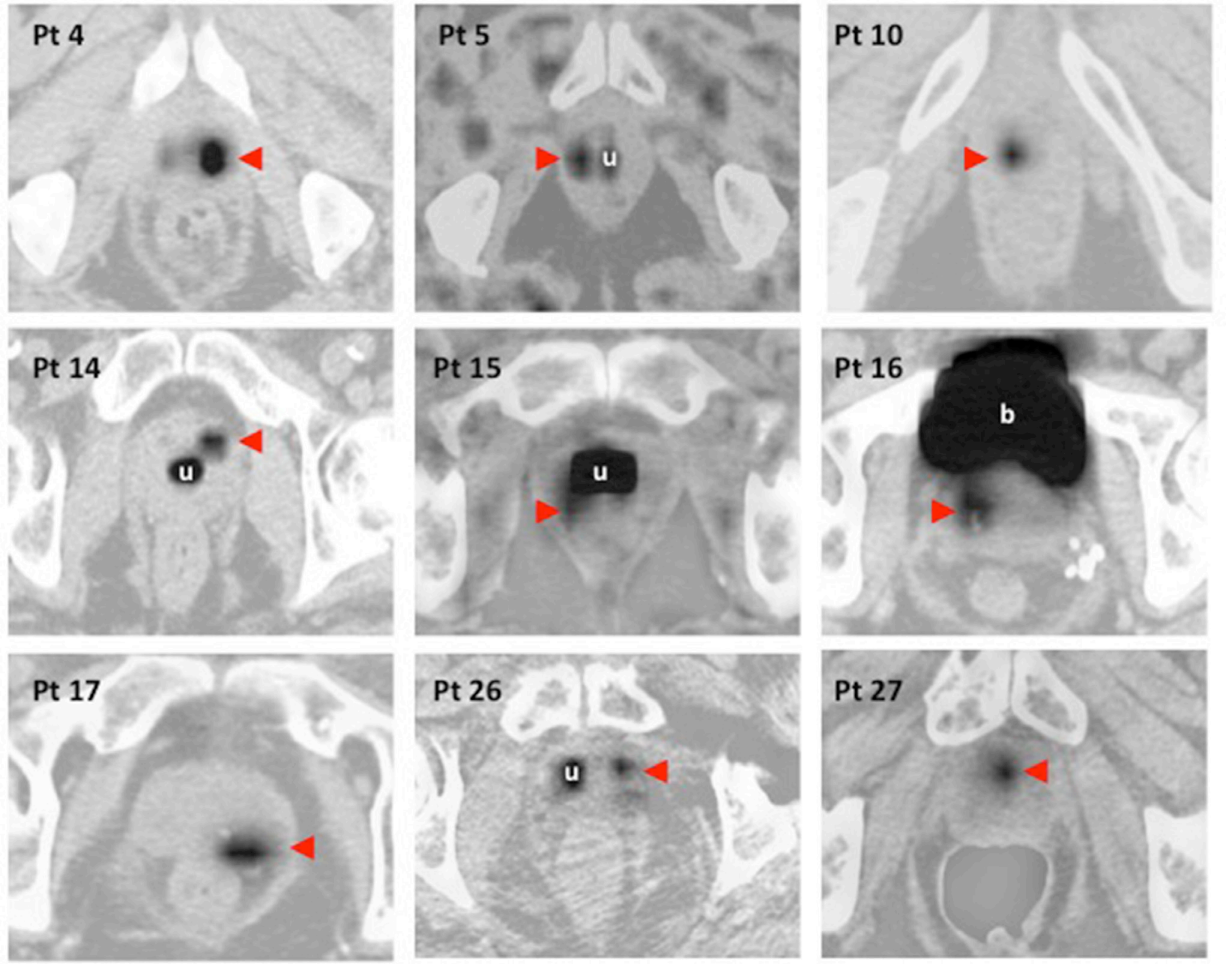
Native FMISO uptake images and attenuation-corrected FMISO images fused with CT-scan images Eleven FMISO-positive regions (red arrows) were determined in 9 out of 27 patients. u: urethra; b: bladder. In patient 5 and 26, only one FMISO-positive region is shown.

**Figure 2:**
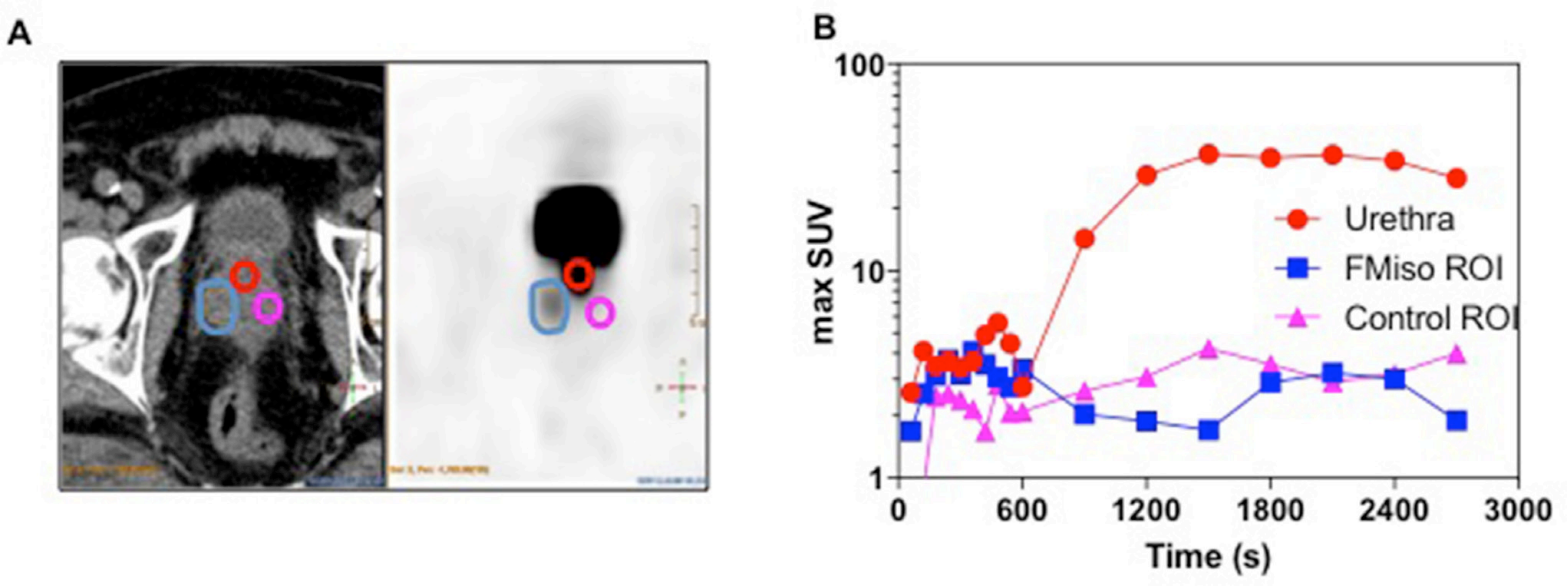
Dynamic images analyses in three FMISO-positive patients with a past history of TURP FMISO ROIs were delineated on 3.5h images and reported on 45-minute images where the urethra was also contoured (**A**). A time/activity curve was generated over 45 minutes in patient #26 (**B**).

All patients underwent FMISO PET/CT. An FMISO uptake was detectable in seven and nine patients of 27 at 2.5h and 3.5h respectively (Figure 1). In two patients, two non-adjacent FMISO-positive uptakes were detected. In three patients (#10, #15 and #26) with FMISO-positive uptake and a past history of TURP, we compared dynamic time-activity curves of FMISO-positive ROIs and the urethra (Figure 2A). In these patients, the urethra was enlarged and the proximity of small hypoxic regions and urine may have interfered with the determination of true FMISO-positive regions. Between 600 and 900 seconds following FMISO injection, a rapid increase was noted in the urethra but not in FMISO-positive ROI or in control ROI (Figure 2B). We concluded that these intraprostatic FMISO-positive uptakes did not have the same dynamic profile as the urethra and therefore can be considered as true prostatic tissue.

**Figure 3:**
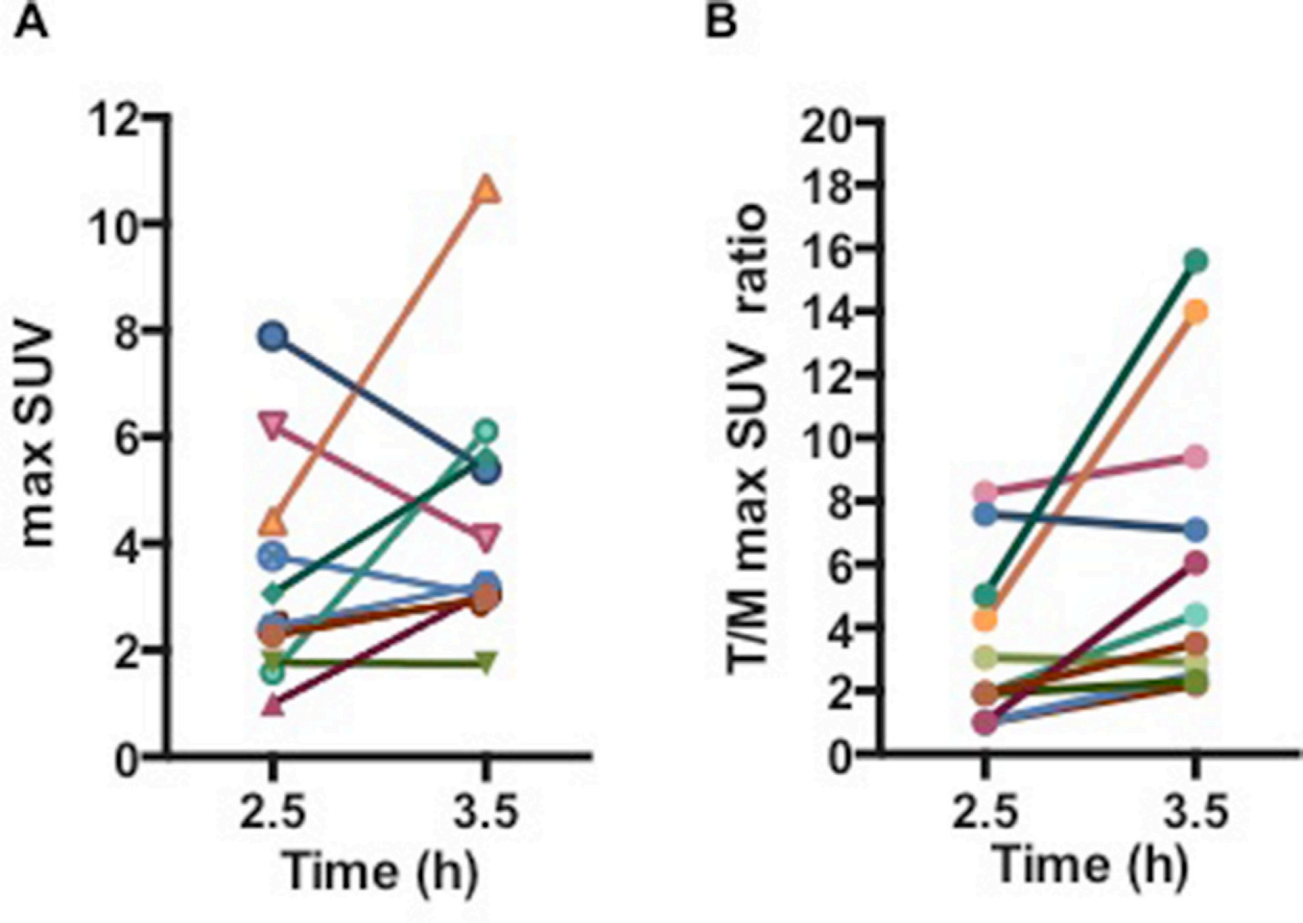
(A) Max SUV and (B) Tumor/Muscle max SUV ratios at 2.5h and 3.5h following 4 MBq/kg injection of FMISO For patients with 2 FMISO-positive volumes, the volumes were summed.

Median FMISO-positive volume was 1.1 ml [0.4–2.4]. Median max SUV and tumor to muscle (T/M) ratios were respectively 3.4 and 3.6 [1.9–8.2] at 2.5h, and 3.2 and 4.4 [2.2–15.6] at 3.5h (Figures 3A, 3B). T/M max SUV ratios were statistically higher at 3.5h than 2.5h after FMISO injection (*p* = 0.0099).

At least one unfavorable intermediate risk factor was present in 78% and 60% of FMISO-positive and FMISO-negative patients (no significant difference). Other characteristics such as age, PSA, Gleason score, prostate gland volume, GTV MRI or GTV FCH volumes did not differ significantly between FMISO-positive and FMISO-negative patients.

### Tissue biopsies and immunochemistry

All patients had diagnostic biopsies. At least one diagnostic biopsy sample was available for hypoxia staining for 24 of the 27 patients. Weak to moderate membranous and/or cytoplasmic expression of Glut1 was observed in cancerous areas in eight patients (one FMISO-positive; seven FMISO-negative) as well as in normal prostate areas in five patients (one FMISO-positive; four FMISO-negative) (Supplementary Figure 1 and Table 1).

**Table 1:** Glut1 expression in prostatic normal and cancer tissues in FMISO-positive and FMISO-negative patients

Patient ^#^	Normal tissue			Adenocarcinoma		
	cyt	mb	%	cyt	mb	%
FMISO-positive						
4	+	++	10%	-	-	
5						
10	-	-		-	-	
14	-	-		-	-	
15	-	-		-	-	
16	-	-		+	-	10%
17	-	-		-	-	
26	-	-		-	-	
27	-	-		-	-	
						
FMISO-negative						
1	-	-		-	-	
2	-	-		+	-	10%
3	-	-		-	-	
6	-	-		-	-	
7	-	-		++	++	10%
8	-	-		-	-	
9	-	-		-	-	
11						
12	-	-		+	-	100%
13	-	-		-	-	
18	++	++		+	++	50%
19	-	-		-	++	10%
20						
21	-	-		-	-	
22	-	-		-	-	
23	+	++	20%	++	-	100%
24	+	-	100%	++	-	50%
25	++	-	100%	-	-	

The extent of expression of Glut1 (“staining frequency”) was recorded as percentage of the entire tumor sample that stained positive with consideration of staining intensity scored as low “+”, intermediate “++” or high “+++”

### Correlation of FMISO/MRI/Choline/histology

In a first approach to match biopsies and imaging, we roughly defined tumor location on prostate sextants according to pathology reports and anatomical locations on MRI, FMISO and FCH images in FMISO-positive patients (Supplementary Figure 2). Cancer cells were found on biopsy in areas considered tumor-involved on MRI or FCH imaging in 7 and 9 patients respectively. Glut1 positive regions were located within regions containing prostate cancer cells in 2 out of 2 cases, but neither of the two Glut1 positive biopsies were located at at a site of FMISO uptake. Parts of the FMISO foci were located in the same sextant of the biopsies containing tumor cells, Glut1 positive cells, MRI and FCH in 5, 0, 4 and 9 patients out of 9 respectively (Supplementary Table 2).

In a second approach, we fused images (Supplementary Figure 3). A weak correlation was observed between FMISO and FCH or MRI-positive areas (median Dice coefficient = 0.01 and 0.03 respectively; Table 2). FMISO volumes did not intersect at all with MRI or FCH in one and three patients respectively (Supplementary Table 2). In the remaining patients, a median percentage of 25% (range 17%–65%) and 45% (range 11%–82%) of FMISO volume intersected with FCH or MRI volumes.

**Table 2:** Fusion of FMISO with MRI and FCH volumes

Patient	Dice Index FMISO &			Intersect FMISO &	
		FCH	MRI	FCH	MRI
4		0	0	0 %	0 %
5		0.01	-	50 %	-
10		0.02	-	17 %	-
14		0.02	0.02	25 %	50 %
15		0.22	0.12	30 %	45 %
16		0	0.03	0 %	40 %
17		0.13	0.20	65 %	82 %
26		0.00	0.03	0 %	50 %
27		-	0.02	-	11 %

Overlap is expressed as percentage of FMISO volume overlapping with MRI or FCH volume. In one patient, FCH PET was not performed for technical reasons. MRI was contra-indicated in two patients with pacemakers.

### Patient outcomes following radiotherapy

In an exploratory analysis, we retrospectively assessed biochemical relapse-free survival. After a median follow-up of 35 months, 1 out of 18 and 4 out of 9 patients relapsed in the FMISO-negative and positive cohorts respectively (*p* = 0.29; Supplementary Figure 4). Using FCH PET, the site of relapse was defined as: intra-prostatic (one FMISO-negative patient and two FMISO-positive patients), pelvic lymphnode (one FMISO-positive patient) and unknown (one FMISO-positive patient). In the two FMISO-positive patients who relapsed after radiotherapy, FCH PET-defined relapses did not intersect with initial FMISO-positive regions. Both relapses were located at a distance of 5 and 14 mm respectively from the edge of the initial FMISO-positive region.

## DISCUSSION

Since hypoxia is associated with aggressiveness and radioresistance in prostate cancer, mapping hypoxia within the prostate using functional imaging may help personalize the treatment of prostate cancer patients. Our pilot study is the first to show that FMISO PET/CT can be used to detect hypoxia within the prostate. We found FMISO-positive volumes in both normal and cancerous areas of the gland. Glut1 staining can also be distributed between normal and cancerous areas, as hypoxia may occur in either normal or cancerous areas

Because hypoxia is an important prognostic factor, many groups have tried to evaluate the hypoxic fraction of prostate tumors. The presence of hypoxia was proven by direct oxygen measurements using Eppendorf pO_2_ microelectrodes [6, 12], and the direct measurement of low oxygen concentrations [5] or the presence of intrinsic hypoxia markers. In a small series of four prostate cancer patients at different stages (localized, locally advanced and metastatic), a FMISO-positive uptake was detected in three patients with hypoxic fractions of 15.7, 20.7 and 93.9% [11]. More recently, FMISO was used in a high-risk prostate cancer patient [13]. The non-invasive FMISO imaging results of our pilot trial confirm these previous reports in a homogenous series of 27 intermediate-risk only prostate cancer patients undergoing radiotherapy. We did not confirm our original hypothesis since only one-third of patients showed some hypoxia. This proportion is however comparable to the proportion of hypoxic patients with a score 4 HIF-1 alpha staining (27%) in a large radiotherapy cohort of localized prostate cancer patients [3]. FMISO PET has largely been used in many kinds of human cancer to detect hypoxia in a non-invasive manner [10], though data in prostate cancer are very limited. These FMISO volumes did not correspond to urine-contaminated regions within the prostate, even in patients with a past history of TURP. Conversely, ^18^F-FAZA PET was unable to detect hypoxia in a series of 14 patients undergoing prostatectomy [14]. There is no clear explanation for this difference. In both series, median PSA was similar (8 ng/ml) but our patients were older (mean age: 76 vs. 60.6) and presented with larger tumors (mean FCH GTV 11 cm^3^ *vs.* mean tumor volume 4 cm^3^) than in the FAZA series. It is possible that older patients may have more hypoxic prostate tissue, and that larger tumor volumes are more likely to be hypoxic compared with smaller volumes.

It was not possible to correlate hypoxia evaluation using Glut1 staining and FMISO images within the prostate. Intrinsic markers that report on hypoxia-induced molecular events (e.g., HIF-1α, GLUT1, CAIX, osteopontin expression) rather than hypoxia itself have been employed as markers of tumor oxygenation with a variable specificity [3, 14, 15]. We selected Glut1, a downstream target of HIF-1α in the hypoxia response pathway, since its staining correlated with pimonidazole in a prostatectomy series [15]. In our series, we found some positivity for Glut1 staining, both in malignant and non-malignant prostate regions, but in a much lower proportion of patients (10/27). Glut1 expression in prostate cancer is highly variable with some reports showing a low to very low frequency of Glut1 immunostaining [16, 17] and a high to very high frequency in others [18, 19], with no relationship with Gleason score, T stage or PSA. An FMISO volume was not detected in 8 patients with some degree of Glut1 expression. Hypoxic prostate volumes are often very small [20, 21] and PET may be technically unable to detect volumes of diameter less than 3 mm given that its spatial resolution is about 4 mm. The FMISO images revealed small volumes (less than 2.5 cm^3^). Moreover, although immunohistochemistry markers such as Glut1 give information about hypoxia, they do not measure absolute O_2_ concentrations. Conversely, an FMISO volume was detected in 7 patients without Glut1 expression on diagnostic prostate biopsies. One possibility is that FMISO signal is falsely positive in these patients. Another possibility is that prostate biopsies might miss small intraprostatic hypoxic areas. Correlating FMISO images with hypoxia markers would require a surgical study in which patients received the hypoxic radiotracer and an extrinsic marker such as pimonidazole prior to prostatectomy.

At least one hypoxic subvolume was located within the dominant intraprostatic lesion (DIL) as defined on FCH PET or MRI in the majority of FMISO-positive patients (eight of nine). Similarly, the FMISO volumes were located to the same prostate sextant as diagnostic prostate biopsies in a majority of patients. This small overlap between imaging modalities may signify that rigid coregistration of combined modality images of small hypoxic regions with anatomic or functional images is problematic because the prostate is highly mobile and deformable. However these findings may also suggest that part of the DIL harbors hypoxic cells and that some part of the histologically normal prostate also harbors hypoxic foci. FMISO-positive regions within the normal prostate may also represent hypoxic areas within smaller satellite tumor foci. Indeed, prostate cancer is a very heterogeneous disease and multiparametric MRI imaging may miss small ( < 0.5 cm^3^) satellite regions [22]. Similarly, FCH PET may miss up to 50% of prostate tumor foci [23]. FMISO-positive regions within the normal prostate may also represent true hypoxic normal gland. Previous studies using oxygen electrode [24], or extrinsic markers of hypoxia such as pimonidazole revealed that hypoxia was found in non-tumor and tumor regions in a large majority of patients [15, 20, 21]. Since hypoxia is suspected to favor genomic instability leading to prostate cancer progression [25], the detection of FMISO-positive regions within the normal prostate might be of interest in monitoring patients with a genetic risk of developing aggressive prostate cancer.

To test the hypothesis that the presence of FMISO volumes might predict for biochemical relapse, we conducted an exploratory analysis and found more biochemical relapse in the FMISO-positive patients than in the FMISO-negative patients, but this difference did not reach statistical significance. Our patient population was not powered enough to be able to assess differences in outcome, which might explain the lack of statistical significance. Moreover, follow-up is still too short to provide a definitive conclusion. Only a specific study focusing on patient outcomes in a larger patient population would be able to test the hypothesis that the presence of FMISO-positive volumes represents a novel prognostic factor. Relapse in the FMISO-positive cohort was intra-prostatic in 2 out of 4 patients. Intra-prostatic hypoxia favors radioresistance [3] and DIL are considered as the major source of local recurrence following radiotherapy [26]. Boosting the radiotherapy dose to imaging-defined DIL is a major strategy to decrease radiation resistance in current dose-painting protocols [27]. Our results suggest that boosting FMISO-defined hypoxic biological target volume (BTV) within the prostate is a feasible approach. Hypoxia also favors a metastatic phenotype. One of the four relapses in the FMISO-positive cohort was detected in a metastatic pelvic lymphnode. Several hypoxia targeting drugs are currently under development and prostate cancer represents a welldefined setting for these molecules [9, 28]. FMISO might help define imaging surrogates of the efficacy of these drugs alone or combined with irradiation.

To conclude, we were able to show that a small ^18^F-fluoromisonidazole-positive volume can be detected in one third of intermediate-risk prostate cancer patients undergoing radiotherapy, which may help personalize treatment decisions for intermediate-risk prostate cancer patients.

## MATERIALS AND METHODS

### Patient selection and follow-up

This prospective study (NCT01898065) was approved by the local Ethics Committee. Written consent was obtained from all participants. Inclusion criteria were NCCN-defined intermediate-risk prostate cancer patients (*i.e.* Gleason 6, PSA 10–20 ng/ml; or Gleason 7, PSA < 20 ng/ml; T < T3) in whom high-dose radiotherapy (> 74 Gy 37 f) to the prostate was indicated. We excluded patients receiving, or having received, hormonal treatment. Following radiotherapy, patients were clinically assessed and had their PSA concentration measured every 6 months. Time to biochemical relapse was defined as time from initiation of radiotherapy to documented PSA relapse according to the Phoenix criteria [29]. The authors declare no conflict of interest regarding this study.

### Imaging modalities

#### ^18^F-fluoromisonidazole

Prior to radiotherapy, all patients underwent a FMISO (IASON Gmbh, Graz-Seiersber, Austria) PET/CT using a Siemens Biograph mCT40 (Knoxville, TN, USA). For all patients, acquisition started immediately after intravenous injection of ^18^F-fluoromisonidazole (3 MBq/kg) gathering dynamic PET images on the pelvis over 45 min on list mode. Pelvic PET images were acquired 2.5 and 3.5 hours after injection, with an acquisition time of 10 min. Low dose CT in the supine position was performed for localization and attenuation correction. Quantification consisted of determining the maximum standard uptake value (SUV) and tumor/muscle SUV (SUV-TM) of FMISO for each volume of interest on PET scanning at 2.5 and 3.5 hours, similarly to cervix cancer [30]. Regions of interest (ROIs) were drawn over the *Gluteus Minimus* muscle and the hottest areas of FMISO uptake. A tumor-to-muscle-ratio threshold of 1.4 was chosen to identify the high FMISO uptake regions, similarly to other published studies [13, 31, 32].

### Dynamic analyses

We analyzed dynamic FMISO PET images using Intellispace Portal ^®^ (PhilipsBV, The Netherlands) to compare the uptake in the urethra and the FMISO-positive regions of interest (ROI). FMISO ROIs were delineated on 3.5h images and reported on 45-minute images where the urethra was also contoured. A time/activity curve was generated over 45 minutes.

### Choline

All patients underwent ^18^F-Choline-PET (FCH, IasoCholine, Iason GmbH, Austria). Patients were examined using a 20 cm axial field of view, a time of flight feature and an in-plane resolution of 4.4 mm in full width at half maximum. All patients were fasted for at least 6 hours before FCH PET/CT. Acquisition commenced one minute after intravenous injection of ^18^F-Choline (3 MBq/kg) with dynamic PET images of the pelvis captured over ten minutes (1 min/frame) to overcome the effect of bladder filling. One hour after injection, a whole body PET/CT was performed.

### MRI

Prior to radiotherapy, all patients underwent a trans-pelvic coil 1.5 T MRI (AERA, 1.5T, Siemens) in the supine position with Gadolinium injection using a surface coil. Axial T2-weighted imaging was performed. Images were reconstructed in a voxel dimension of 0.31× 0.31 × 4 mm^3^. Isotropic axial diffusion-weighted scans were performed using a single-shot echo-planar imaging sequence. Prostate tumor volume was segmented manually by a single expert based on T2W hyposignal. Diffusion and perfusion images were used to help detect the tumor regions, but not for delineation or image fusion. Tumors were classified according to the Prostate Imaging and Reporting and Data System (PI-RADS™) [33]. Since 95 % of all prostate tumor cells will be located within a 5 mm rim beyond the volume detected on MRI [34], we expanded the treatment contour at the non-capsular margin by 5 mm to define the Gross Tumor Volume (GTV MRI).

### Planning CT

Patients were asked to empty the rectum and bladder and then drink 500 ml of water 60 minutes prior to the planning CT scan (BigBore, Philips). Patients were scanned (3 mm-thick slices every 3 mm) in the supine position with conventional head and knee support.

### PET uptake delineation

All uptakes in FMISO and ^18^F-Choline images were semi-automatically delineated using the fuzzy locally adaptive Bayesian (FLAB) algorithm, developed specifically for PET image segmentation [35, 36]. The same process was used for both ^18^F-Choline and FMISO PET images.

### Image coregistration

Image coregistration of FMISO, FCH, MRI and planning CT images was performed using a rigid, non-parametric, affine transformation (IPlan RTimage 4.1, Brainlab AG, Feldkirchen, Germany). To take into account bladder and rectal filling, automatic fusion was adjusted manually based on outer prostate volume and intraprostatic calcifications. To determine whether the FMISO volume was similar to FCH and MRI volumes, a DICE index (DICE = 2 FMISO vol Ω FCH or MRI vol / (FMISO vol + FCH or MRI vol) was calculated. The DICE index is a quotient of similarity between two volumes and ranges between 0 (no similarity) and 1 (complete similarity).

### Immunohistochemistry

The formalin-fixed, paraffin-embedded prostate biopsy histopathology was evaluated for the presence of Glut1 (clone RP128, dilution 1:300, Diagnostic Biosystems), and PIN cocktail with P63 (M7247, dilution 1:50, DakoCytomation) and racemase P504S (M3616, dilution 1:200, DakoCytomation). A single pathologist blinded to imaging results performed the quantitative assessment of protein expression. The extent of expression of Glut1 was recorded as percentage of the entire tumor sample that stained positive, and staining intensity scored as low “+”, intermediate “++” or high “+++” (Supplementary Figure 1).

### Sample size determination and statistical analysis

In a small study, a FMISO-positive volume was detected in 3 out of 4 patients (75%; CI 19.4%-99.4%)[11]. In order to be able to confirm a 75% rate of FMISO-positive patients with a 95% confidence interval ranging between 50.9% and 91.3%, we had to recruit a minimum of 20 patients. Because of the large uncertainties of the hypothesis, we decided to recruit 27 patients in a pilot trial.

Results are expressed as mean values of parameters ± SD. Group differences were assessed using the Wilcoxon signed rank test. Parameters were correlated using a nonparametric test (Spearman rank correlation); *p* < 0.05 was considered statistically significant. Survival curves were compared using a log-rank (Mantel-Cox) test. Statistical tests were performed with Stata 13.1 (Statacorp LP, College Station, TX, USA).

## SUPPLEMENTARY MATERIALS FIGURES AND TABLES



## Abbreviations

FMISO: ^18^Fluoro-Misonidazole
FCH: ^18^Fluoro-Choline
FLAB: fuzzy locally adaptive Bayesian
