# Supplementary material for: Evaluation of tumor hypoxia prior to radiotherapy in intermediate-risk prostate cancer using 18F-fluoromisonidazole PET/CT: a pilot study
Source: Oncotarget. 2018 Jan 13;9(11):10005–15. doi: 10.18632/oncotarget.24234 (PMC5839367; doi:10.18632/oncotarget.24234)
Supplement: Supplementary file 1 [file oncotarget-09-10005-s001.pdf]

Evaluation of tumor hypoxia prior to radiotherapy in intermediate-risk prostate cancer using 18F-fluoromisonidazole PET/CT: a pilot study

SUPPLEMENTARY MATERIALS

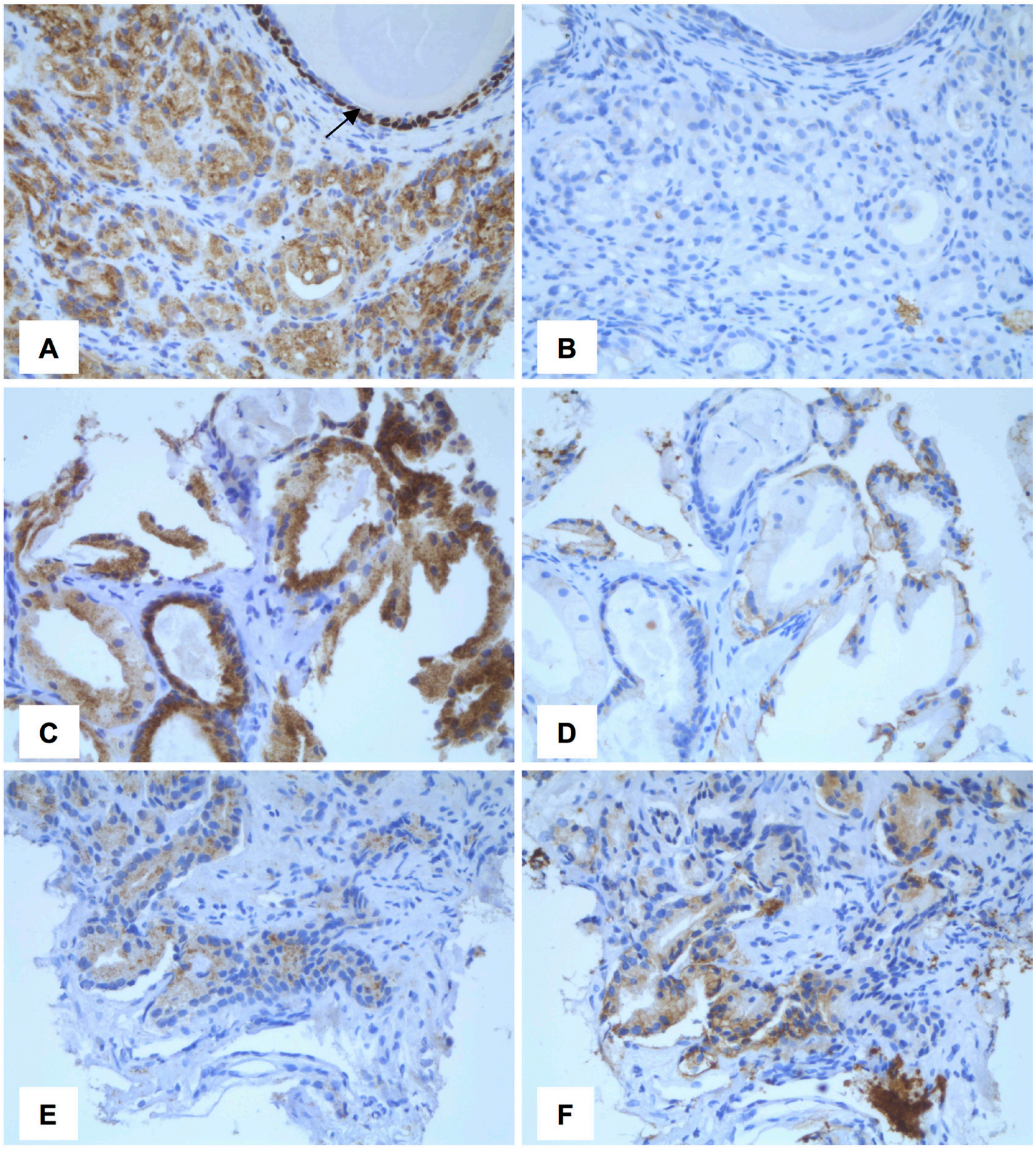

Supplementary Figure 1: Representative images (x20) of Glut1 staining: negative, membrane and cytoplasm positive prostate cancer glands. (**A**) Intense cytoplasmic staining of P504S without nuclear staining of P63 in tumor glands unlike the normal glands (arrow). (**B**) Absence of Glut1 expression in normal and tumor glands; noteworthy membranous and cytoplasmic staining of red blood cells which constitutes an excellent internal control. (**C**) Intense cytoplasmic staining of P504S of tumor glands without nuclear staining of P63. (**D**) Membrane staining of Glut1 of tumor glands. (**E**) Moderate cytoplasmic staining of P504S without nuclear staining of P63 of tumor glands. (**F**) Membranous and cytoplasmic staining of Glut1 of tumor glands.

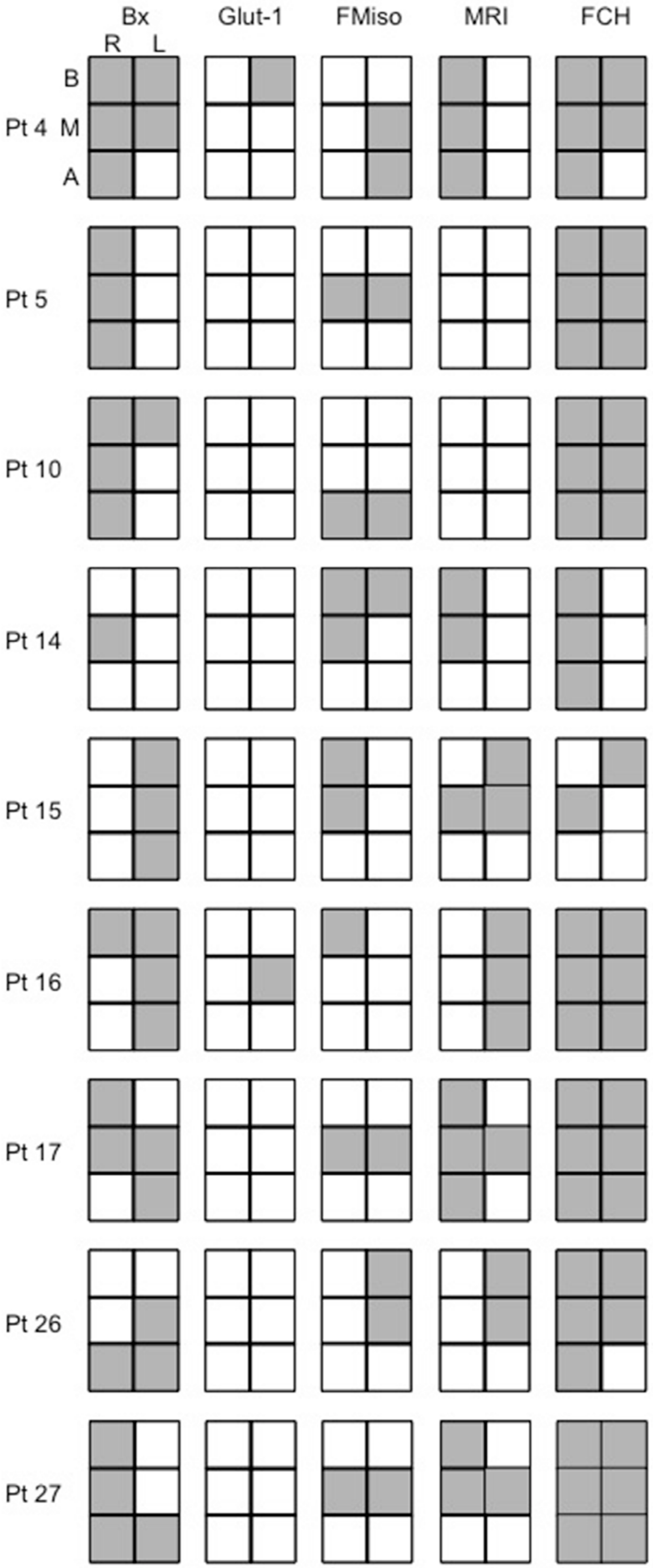

Supplementary Figure 2: Colocalization of FMISO-positive images, hypoxia and prostate cancer. The prostate was divided in six sextants (3 levels: Apical -A-, Median -M- and Basal -B- and 2 sides Right -R- and Left -L-). In nine patients with a FMISO-positive image, tumor location was roughly defined on prostate sextants according to diagnostic biopsies (Bx), Glut1 staining for hypoxic regions (Glut1), FMISO PET/CT images prior to radiotherapy, diagnostic MRI and ^{18}F- (FCH).

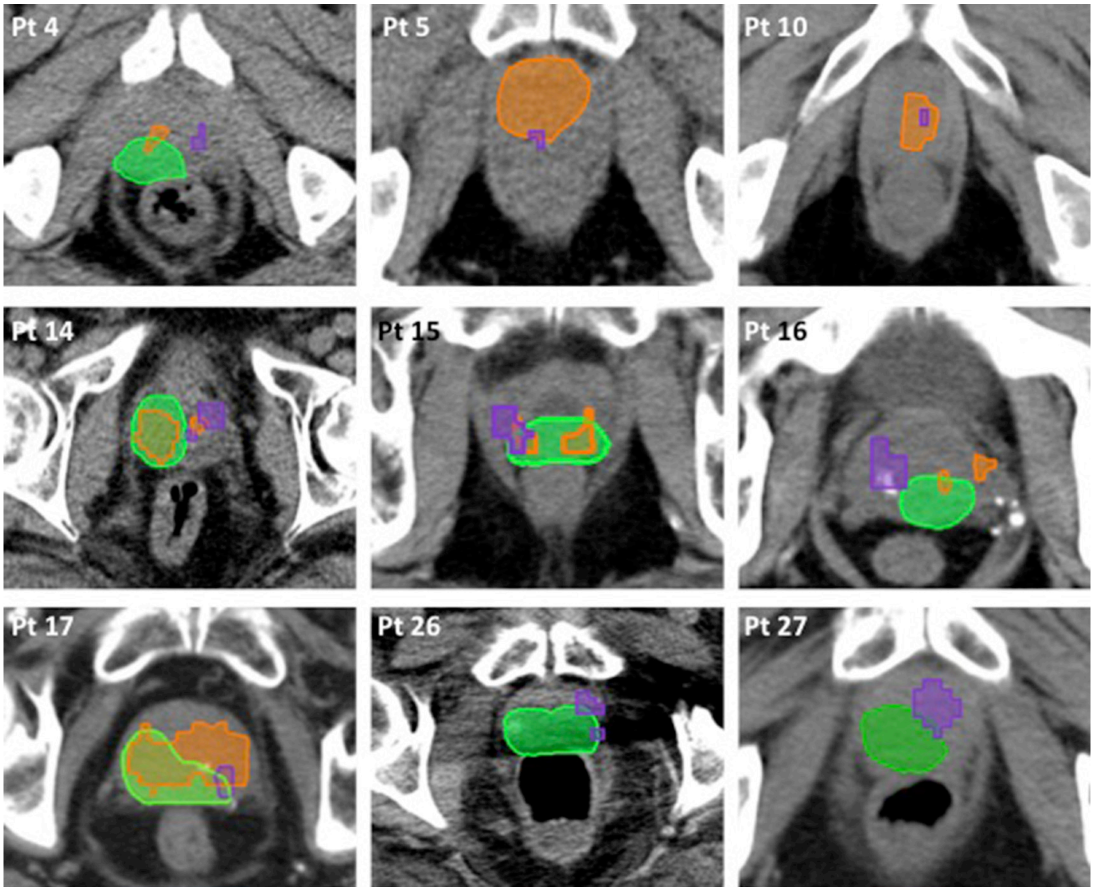

Supplementary Figure 3: Rigid coregistration of FMISO GTV (purple) images with MRI GTV (green), FCH GTV (orange) in all FMISO-positive prostate cancer patients.

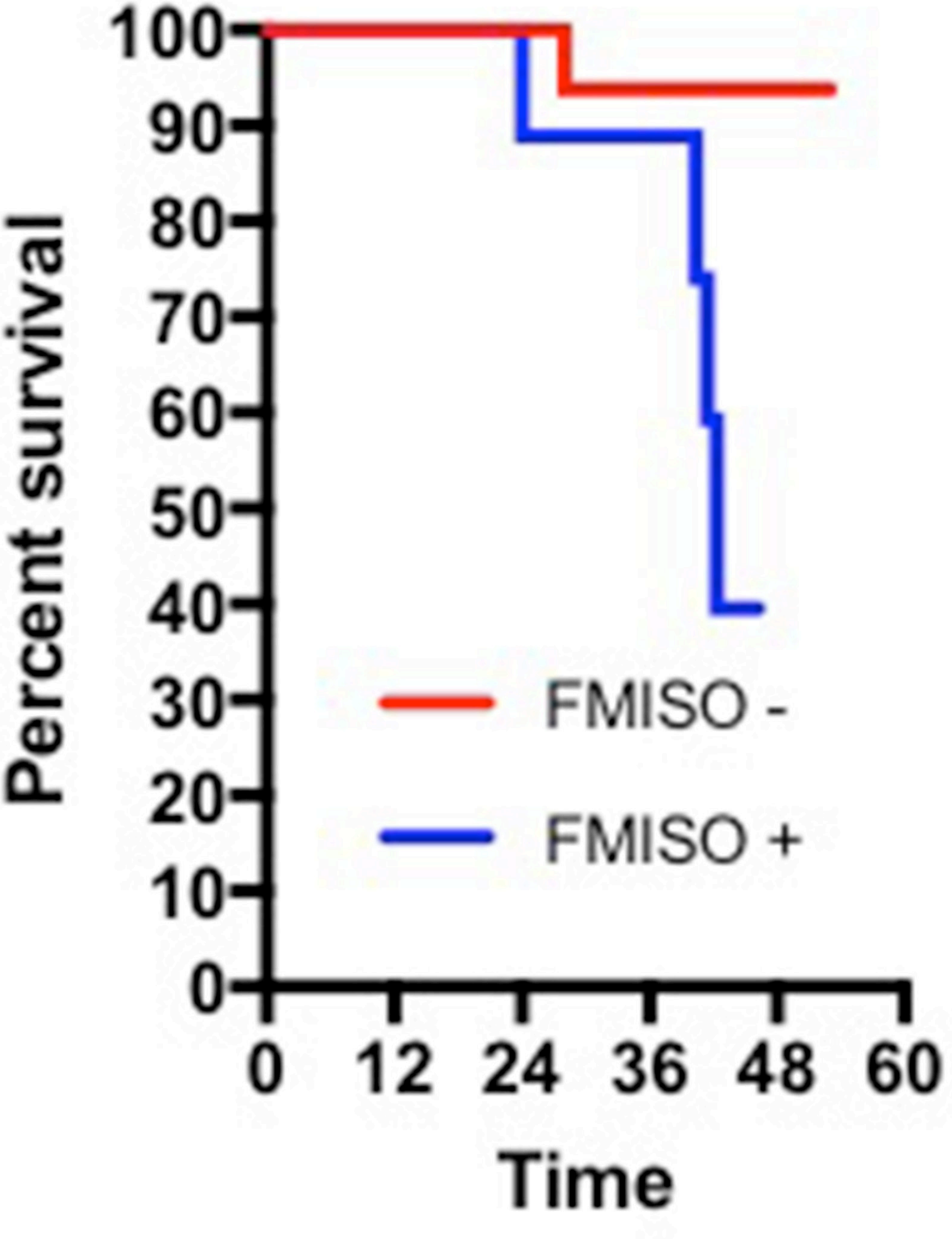

Supplementary Figure 4: Biochemical relapse-free survival following radiotherapy in the FMISO-positive (blue) and FMISO-negative (red) patients.

Supplementary Table 1A: Characteristics of FMISO-positive patients

Patient	Age	TURP	PSA (ng/ml)	Gleason score	T stage	# Positive biopsy /total	Pi Rads score
4	74		8.8	7 (3 + 4)	T2a	9/12	5
5	78		6.4	7 (4 + 3)	T1c	7/13	-
10	77	+	13.3	7 (3 + 4)	T1c	11/20	-
14	67		8.5	7 (3 + 4)	T1c	2/11	5
15	76	+	3.5	7 (3 + 4)	T1c	4/12	5
16	81		8	7 (4 + 3)	T2a	7/12	5
17	65		4.5	7 (4 + 3)	T1c	4/8	5
26	80	+	11	6 (3 + 3)	T1c	3/12	4
27	75		6.5	7 (3 + 4)	T2a	8/13	4

SupplementaryTable 1B: Characteristics of FMISO-negative patients

Patient	Age	TURP	PSA (ng/ml)	Gleason score	T stage	# Positive biopsy /total	Pi Rads score
1	76	+	6.7	7 (4+3)	T2a	4/12	5
2	77		15.1	7 (3+4)	T2c	6/12	5
3	75		2.2	6 (3+3)	T2c	6/7	3
6	72		5.4	7 (4+3)	T1c	5/12	-
7	77		19	7 (3+4)	T1c	2/11	5
8	76		7.2	7 (4+3)	T2b	4/15	5
9	74		3	7 (3+4)	T2a	4/14	3
11	78		14.6	6 (3+3)	T1c	4/19	2
12	76		8.5	6 (3+3)	T2b	5/16	5
13	78		7.8	7 (3+4)	T1c	12/12	4
18	76		4.6	7 (3+4)	T1c	4/12	5
19	78		7.8	7 (3+4)	T2a	3/12	5
20	68		10.2	7 (3+4)	T2a	6/6	5
21	76		11	7 (4+3)	T1c	14/28	5
22	72		5.4	7 (4+3)	T2a	5/12	5
23	58		7.4	7 (3+4)	T1c	4/12	4
24	75		11.7	7 (3+4)	T1c	3/12	2
25	68		14.6	7 (3+4)	T1c	5/12	5

TURP: Trans-urethral resection of the prostate.

Supplementary Table 2: Correlation of FMISO/MRI/Choline/histology

	Tumor cells	Glut-1 positive cells	MRI- positive	F-Choline positive
FMISO signal within the same sextant	5/9	0/8	4/7	9/9
FMISO signal intersects with images			6/7	6/9
